# The Double-Edged Sword of Autoimmunity: Lessons from Multiple Sclerosis

**DOI:** 10.3390/toxins2040856

**Published:** 2010-04-22

**Authors:** Anne Lise K. Hestvik

**Affiliations:** Institute of Immunology, Faculty of Medicine, University of Oslo, Oslo University Hospital, Rikshospitalet, 0027 Oslo, Norway; Email: anne.lise.hestvik@rr-research.no; Tel.: +47-23073767; Fax: +47-23073510

**Keywords:** autoimmunity, multiple sclerosis, T cells, B cells, glatiramer acetate

## Abstract

The relationship between immune responses to self-antigens and autoimmune disease is unclear. In contrast to its animal model experimental autoimmune encephalomyelitis (EAE), which is driven by T cell responses to myelin antigens, the target antigen of the intrathecal immune response in multiple sclerosis (MS) has not been identified. Although the immune response in MS contributes significantly to tissue destruction, the action of immunocompetent cells within the central nervous system (CNS) may also hold therapeutic potential. Thus, treatment of MS patients with glatiramer acetate triggers a protective immune response. Here we review the immunopathogenesis of MS and some recent findings on the mechanism of glatiramer acetate (GA).

## 1. Introduction

Autoimmunity can be defined as an adaptive immune response directed against the body’s own tissues. At the beginning of the twentieth century, the German physician Paul Ehrlich coined the term “horror autotoxicus” arguing that the normal body would never mount an immune response against its own tissue. According to this view, any autoimmune reaction was destructive and connected to human disease. We now know that the causal relationship between autoimmune reactions and autoimmune diseases is more complex. 

It is estimated that about 20–50% of the T cell and B cell receptors created during receptor gene recombination are self-reactive [1]. Although most T and B cells carrying such self-reactive receptors are deleted during maturation, a high frequency of autoreactive T cells, B cells and autoantibodies is present in the normal repertoire without causing disease. Indeed, it is proposed that recognition of self is essential for survival of naïve lymphocytes and that it can enhance reactivity to foreign antigen [2], that it may regulate the extent and duration of immune responses [3], and that autoantibodies can contribute to the clearance of damaged tissue [4]. Furthermore, recognition of self-proteins in the absence of costimulation is important for the maintenance of immunological tolerance [5].

### 1.1. Protective autoimmunity in the central nervous system

Studies in rodents have demonstrated an essential role for regulatory T cells in the reduction of injury after stroke [6,7]. On the other hand, observations in a model for crush injury to the optic nerve have shown that the same myelin basic protein (MBP) specific T cells that cause experimental autoimmune encephalomyelitis (EAE) in mice also can have a protective function by reducing secondary degeneration of neurons after primary injury to the optic nerve or spinal cord [8,9]. This effect was shown to be specific for T cells reactive with central nervous system (CNS) antigens, as T cells with other specificities did not confer protection even though they were shown to home to the injury site [8]. A follow-up study demonstrated that suppression of the autoimmune reaction by tolerance induction to MBP or by the injection of CD4^+^CD25^+^ regulatory T cells reduced the protective effect of MBP specific T cells [10]. Thus, a degree of autoimmunity was required to confer protection. These observations have implicated T cells as important mediators of CNS neurogenesis, the process by which neuronal precursor cells give rise to new neurons in certain areas of the brain [11,12]. Accordingly, it was shown that T cell deficient mice displayed decreased neurogenesis from endogenous precursor cells compared to normal mice, and that this could be partly restored by reconstitution of the T cell pool [13]. 

The protective effect of CNS-reactive T cells most likely involves activation of local antigen presenting cells (APCs) present at the injury site [14,15]. It has been demonstrated that activation of microglia by either the Th1 cytokine IFN-γ or the Th2 cytokine IL-4 induced neuronal and oligodendroglial differentiation from adult neuronal precursor cells [16], and thus hypothesized that the release of cytokines may serve a function in the recruitment of neuronal precursor cells to the injury site. In support of this, neuronal precursor cells layered onto hippocampal slice cultures were shown to migrate towards sites treated with inflammatory stimuli, such as TNF-α or IFN-γ [17]. This was dependent upon cytokine-induced upregulation of chemo-attractants. Differentiation of progenitor cells arriving at the injury site may in part be mediated by growth factors. Activated microglia and macrophages have been shown to secrete a variety of growth factors *in vivo* [18], and may reciprocally induce the production of growth factors in other cells [13]. Furthermore, activated human T cells, B cells and monocytes in inflammatory MS lesions as well as myelin oligodendrocyte glycoprotein (MOG) reactive T cells from healthy individuals express nerve growth factors [19,20]. 

### 1.2. Autoimmune diseases

The relation between benign autoimmunity and the progression and establishment of an autoimmune disease is unclear. An autoimmune disease can be organ specific, such as in type 1 diabetes and multiple sclerosis (MS) or systemic such as in systemic lupus erythematosus and Sjogren’s syndrome and is characterized by a chronic adaptive immune response directed against self-tissue. About 3–5% of the world’s population is affected by an autoimmune disease with women accounting for 78% of cases [21]. It is believed that a combination of risk-associated polymorphisms in immunoregulatory genes, infectious agents and other environmental triggers contribute to the initiation and propagation of disease [22,23]. Thus, a few examples of human autoimmune diseases induced by defined microbes exist, such as rheumatic fever and Guillain-Barré syndrome. These are thought to arise from antigenic mimicry between the infectious agent and human tissue triggered during the initial inflammatory response. For the majority of human autoimmune diseases, however, the link to infection remains circumstantial. This may be due to the time lag between infection and establishment of disease and the potential contribution of subclinical infections. 

## 2. Immune Surveillance of the Central Nervous System

The CNS is comprised of the brain and spinal cord, surrounded by three layers of meningeal membranes. The blood brain barrier (BBB) is a feature of the cerebral vasculature, which restricts access of ions and other solutes present in the blood into the brain parenchyma. The anatomical structure of the BBB comprises two cell layers, which are separated by the perivascular space. One is formed by endothelial cells lining the brain capillaries and an underlying basement membrane, and the other is formed by astrocytic foot processes and their parenchymal basement membrane. Unlike other tissues, the endothelial cells of the BBB display no fenestration and are connected by tight junctions, which efficiently restrict the traffic of molecules and cells in and out of the brain. The cerebrospinal fluid (CSF) bathes the brain and is produced from arterial blood by the choroid plexus. It flows from the ventricles of the brain into the subarachnoid space located between the arachnoid and the pial membrane and is eventually absorbed into the venous circulation. The CSF communicates with the interstitial fluid of the brain through the perivascular spaces. Due to the lack of tight junctions in the ependymal linings of the ventricles, small hydrophilic molecules as well as proteins diffuse freely between the CSF and brain interstitium (reviewed in [24]).

Under physiological conditions, immune cells enter the CNS at a very low level for the purpose of immune surveillance [25,26]. In contrast, during inflammatory diseases such as MS, activated cells readily traverse the inflamed BBB [27]. CNS fluids continuously drain into cervical lymph nodes, ensuring communication with the peripheral lymphoid system, but the absence of secondary lymphatic structure, the low expression of major histocompatibility complex (MHC) class II molecules and the lack of dendritic cells (DCs) in the CNS have questioned how immune surveillance of the brain takes place under physiologic conditions [25,26].

Perivascular cells within the subarachnoid space probably play a key role in immune surveillance. It is suggested that activated memory cells enter the CSF from the systemic circulation and monitor the subarachnoid space under physiologic conditions [26]. This is strongly supported by observations in EAE where parenchymal inflammation and disease onset is preceded by inflammation and accumulation of Th17 polarized CD4+ T cells in the subarachnoid space [28,29]. Furthermore, it was recently demonstrated that Th17 cells expressing the chemokine receptor (CCR)6 were allowed access into the perivascular space through the choroid plexus by interaction with the CCR6 ligand, CCL20 [30]. This step triggered and was indispensable for a second wave of inflammation mediated by T cell infiltration through the BBB. CCR6 was found to be constitutively expressed by cells of the choroid plexus also in humans, and the entry of Th17 cells into the CSF was suggested to control immune surveillance of the CNS during physiologic condition. 

### Migration of cells across the blood brain barrier

The migration of mononuclear cells across an inflamed BBB is a two-step process, which first requires entry across the endothelial cell layer and its basement membrane into the perivascular space. Several adhesion molecules, including activated leukocyte cell adhesion molecule, intracellular adhesion molecule, vascular cell adhesion molecule (VCAM)-1, α4-integrin and laminins seem to be selectively involved in the adhesion and transmigration of T cells (reviewed in [31]). Adhesion molecules are believed to aggregate in microdomains on the endothelium, so-called transmigratory cups, which guide the migration of lymphocytes across inflamed cerebral vessels. The monoclonal antibody (mAb) natalizumab, used in the treatment of MS, efficiently inhibits the infiltration of lymphocytes into the brain by blocking the VCAM-1-ligand, α4β1-integrin [31]. Furthermore, T cell transmigration could be selectively inhibited by laminin-α5, an adhesion molecule expressed on the endothelial basement membrane [32]. 

To reach the brain parenchyma from the perivascular space leukocytes must traverse the parenchymal basement membrane and the glia limitans, a thick layer of astrocytic processes that seals the entire surface of the CNS. The molecular mechanisms facilitating this step are less defined, but are thought to involve secretion of matrix metalloproteinases (MMPs) by perivascular macrophages or DCs. Hence, mice were made resistant to EAE by deletion of MMP-2 and MMP-9, and T cells were trapped in the perivascular space [33]. Thus, cells that gain access to the perivascular space through interaction with inflamed brain endothelium may only traverse the glia limitans into the parenchyma if they recognize their cognate antigen presented by perivascular APCs [34,35]. Also the strength of lymphocyte reactivation in the perivascular space may determine migration into the brain parenchyma [36]. MHC expression by endothelial cells may also play a role in the recruitment of antigen specific T cells, as recently demonstrated for the migration of CD8^+^ T cells across the BBB [37]. Finally, T cells that infiltrate the brain parenchyma may interact with resident microglia, which in response to CNS inflammation acquire a macrophage-like phenotype with increased expression of costimulatory and adhesion molecules [38]. 

## 3. Multiple Sclerosis

MS was first described in 1868 by Jean-Martin Charcot [39], but early reports of people suffering distinct neurological symptoms analogous to MS date back to the middle ages [40]. Today MS has a prevalence that generally ranges from 2–150 per 100,000, although this can be significantly higher in certain regions. The etiology of MS is complex and involves genetic and environmental factors [41,42,43].

### 3.1. Clinic

MS usually presents with a clinically isolated syndrome, a neurological episode suggestive of inflammation and demyelination, but not sufficient by itself to qualify for an MS diagnosis. In the majority of patients, disease typically evolves with irregular relapses followed by more or less complete remission. The use of magnetic resonance imaging allows visualization of affected CNS sites. Radiological evidence of inflammation disseminated in time and space is part of the revised diagnostic criteria for MS [44]. At the onset of disease, the majority of patients follow a relapsing-remitting (RR) course, whereas in about 15% of patients, disease progresses without intermittent relapses in what is referred to as primary progressive MS [45]. Progression of disease in most RRMS patients will over time also be devoid of remissions and evolve into a secondary progressive course [46], where the most important clinical feature is the progression of disability in the absence of relapses. 

### 3.2. Etiology

Clustering of MS cases within families and the sharp decline in concordance with increasing genetic distance demonstrate the genetic contribution to MS [41]. The strongest genetic association to MS is found within the human leukocyte antigen (HLA) complex. The HLA-DRB1*1501 allele is thought to confer the primary association in Caucasians and Afro-Americans [47,48]. Lately, genome wide association studies have pointed out an association between MS and immunoregulatory genes encoding IL-2 and IL-7 receptor α chains, which are associated with activation and homeostasis of T cells [49,50]. 

Several observations demonstrate the environmental contribution to MS risk: (i) the relative low concordance rate in monozygotic twins, (ii) the influence on MS risk by migration to areas of low or high MS prevalence, and (iii) the increase in female:male sex ratio observed over time [42,43]. Both infectious and non-infectious factors, such as vitamin D and smoking, have been implicated by epidemiological evidence. It seems that environmental factors in childhood contribute to MS risk and a study on adoptees have demonstrated that environmental factors in MS operate on a population basis and not in the microenvironment [51]. 

Of many possible infectious agents suggested to confer MS risk, Epstein-Barr virus (EBV) is supported by the strongest epidemiological evidence. MS risk is higher in individuals with a past history of infectious mononucleosis, and a temporal increase in serum titres of antibodies to EBV has been shown to correlate with the onset of MS later in life [52]. The functional relevance of EBV in MS is supported by a higher frequency of EBV specific T cell in MS patients [53] and by the demonstration of MBP specific T cells that cross react with EBV-proteins [54,55]. Furthermore, strong CD8^+^ T cell responses to EBV can be detected in cases of early MS [56]. However, the reported identification of EBV-infected B cells in white matter lesion in MS [57] has been challenged by the inability of other groups to reproduce these findings [58,59] leaving it controversial as to whether EBV contributes directly to CNS pathology. 

### 3.3. Pathogenesis

Lessons from EAE have guided much of the research in MS and formed the long-held view that myelin specific CD4^+^ cells play a key role in MS. EAE is caused by a direct attack on myelin proteins mediated by myelin specific CD4^+^ T cells [60]. EAE can be induced in susceptible strains of rodents and nonhuman primates through immunization with myelin proteins or peptides emulsified in complete Freund’s adjuvant or by adoptive transfer of activated myelin specific CD4^+ ^T cells. Although EAE reflects important pathogenic mechanisms in MS, observations such as a dominance of clonally expanded CD8^+^ T cells in active MS lesions, the perpetual intrathecal production of oligoclonal IgG, and the failure to firmly establish myelin proteins as target antigens in MS underscore critical differences in the pathogenesis of human MS and animal EAE [61].

The clinical benefit of blocking migration of lymphocytes into the CNS or of strong immunosuppression with mAbs has for the first time provided conclusive evidence for the detrimental effect of the immune response in MS [62,63]. However, whether the immune response in MS is primarily autoimmune or secondary to neurodegeneration elicited by other factors is still not settled. According to the autoimmune hypothesis of MS, it is assumed that effector CD4^+^ and CD8^+^ T cells are primed in secondary lymphoid organs through antigen specific T cell-APC interactions [64]. In view of recent findings, it may be proposed that reactivation of antigen specific Th17 cells in the perivascular space plays a crucial role for the transmigration of other T cell subsets [29,30]. The upregulation of adhesion molecules and corresponding ligands by brain epithelium and activated lymphocytes allows the migration of activated T cells and B cells across the BBB. It is believed that only those T cells that recognize cognate antigen will be retained in the brain [27]. Reactivation may occur by cross recognition of myelin or neuronal antigens, by recognition of an original infectious agent [57] or of other antigens present in the brain, such as idiotopes on clonally expanded B cells and IgG molecules [65]. Reactivation of T cells triggers parenchymal inflammation, which recruits T cells, B cells, DCs and microglia to the site of inflammation. The release of pro-inflammatory cytokines, direct damage mediated by MHC class I restricted CD8^+^ T cells and indirect damage by MHC class II restricted CD4^+^ T cells, complement deposition and local activation of microglia and macrophages [66], may all have a role in the inflammatory response. The reason why the immune response becomes chronic remains unknown, but could be explained by site-specific expression of autoantigens, persistence of latent infections, a permissive CNS environment or a combination of these factors.

### 3.4. The intrathecal immune response in MS

A hallmark of the immune response in MS is the formation of isolated areas of inflammation called MS lesions. Lesions can appear both in the white and in the grey matter of the brain and are often found around the ventricles, in the optic nerve, in the brain stem and in the spinal cord [67]. Tissue damage involving grey matter and normal appearing white matter can also be observed [68]. Within lesions the most characteristic pathological feature is demyelination. Axonal damage is probably present from early in the disease process and numerous transected axons can be visualized in active lesions [69]. Mononuclear infiltrates of CD4^+^ T and CD8^+^ T cells, B cells and macrophages are present to various extents and are thought to be critical for disease development and progression. Much effort has been devoted to analyzing the phenotype of T and B lymphocytes dominating in the intrathecal compartment as a means to identify potential antigens and to understand the underlying disease process. 

#### 3.4.1. T cells

Within the active lesion clonally expanded CD8^+^ T cells outnumber CD4^+^ T cells, which are more polyclonal and often found in the periphery of the lesion [70,71]. By analyzing 22 tissue blocks from patients and healthy controls, the majority of T cells in active MS lesions were found to express IL-17 [72]. Unlike what is described for EAE, both CD4^+^ and CD8^+^ T cells from MS lesions stained positive for IL-17. Their expression of cytolytic granules and their ability to kill neurons *in vitro* [73] have pointed out Th17 cells as a critical subset of T cell in MS [74].

T cells within MS lesions display a restricted receptor repertoire [75,76], suggesting that a limited number of clones participate in local immune reactions. Furthermore, central memory T cells within MS lesions have been shown to lack CCR7 [77], indicating that they had differentiated into effector T cells upon restimulation with antigen. However, the antigen-specificity of T cells in MS remains unclear. In light of EAE, the search for an MS target antigen has revolved around myelin peptides, but the role of myelin specific T cells in MS is uncertain. The frequency of MBP specific T cells is overlapping in MS patients and controls, although T cells from MS patients seem to display a higher frequency of activation markers and tend to belong to the memory pool of T cells [78]. A phase II clinical trial where MS patients were immunized with an altered peptide ligand based on an immunodominant MBP epitope, resulted in clinical exacerbations associated with an increase in the frequency of T cells specific for the MBP epitope in the CSF [79]. This suggests that MBP specific T cells may cause encephalomyelitis also in humans, but does not pinpoint MBP specific T cells as culprits in MS. In a young patient suffering from hyperacute MS, T cell infiltrates were shown to display reactivity to myelin proteins [80]. However, earlier attempts to chart the specificity of T cell clones isolated from MS lesions have not detected reactivity to MBP or proteolipid protein (PLP) [81]. The pathogenic role of myelin specific T cells is further complicated by the therapeutic potential these cells may have [82].

The search for alternative antigens in MS has suggested the stress protein αB-crystallin as a potential target. αB-crystallin is a small heat shock protein and one of the most abundantly expressed proteins that is found in active MS lesions, but not in normal brain [83,84]. In comparison to a variety of other myelin proteins, which included MBP, PLP and MOG, αB-crystallin elicited strong proliferative responses in peripheral blood lymphocytes from MS patients. Thus, other potential T cell targets in MS may be proteins expressed uniquely in MS brains and not in a normal brain [85]. A recent gene expression study of cortical MS lesions and meninges did, however, only identify over expression of immunoglobulin (Ig) related genes [86].

#### 3.4.2. B cells

Several observations demonstrate that B cells are involved in the disease process of MS. B cells, as well as deposits of Ig and complement, are found within MS lesions [87] and more than 95% of MS patients display a perpetual intrathecal synthesis of oligoclonal Ig, which can be visualized as distinct oligoclonal bands (OCBs) by isoelectric focusing or agarose gel electrophoresis. The OCBs of MS patients are predominantly IgG1. OCBs can also be observed in infectious diseases of the CNS where the antigenic target is the infectious etiologic agent [88,89]. 

Analyses of transcribed BCR V genes from CSF and MS lesions have revealed a population of B cells that is clonally expanded, displays a limited H chain repertoire and that contains numerous replacement mutations (reviewed in [90]). These observations suggest that the B cell response in MS is an antigen-driven T cell dependent process. Prominent clonal expansion of CSF B cells is an early feature of MS, suggesting that antigen-specific B cell responses may be implicated at the onset of disease [91,92]. Short-lived plasma blasts are more prevalent in the CSF than mature plasma cells, and have been suggested to be the main effector B cell population [93,94]. A comparison of the Ig transcriptome of B cells with the corresponding Ig proteome in the CSF of four MS patients established that CSF B cells were at least one possible source of the OCBs [95]. However, OCB formation in CSF may also result from B cell activation within the CNS parenchyma. Efforts to identify the specificity of the main oligoclonal IgG in MS remains a challenge [96]. 

Antibodies with reactivity against myelin proteins can readily be detected in the CSF of MS patients [97,98]. IgG from CNS tissue was found to contain anti-MOG antibodies in seven of 14 MS patients [99], and nine out of 10 antigen binding fragments from clonally expanded CSF B cells from four MS patients recognized MBP [100]. However, the pathogenic relevance of myelin specific antibodies in MS remains uncertain. 

A curious feature of MS is the perpetual intrathecal production of virus specific antibodies [101,102]. These antibodies are typically directed towards measles, varicella zoster, rota and mumps viruses [101,103,104]. They mainly display IgG1 subclass restriction [105] and are also present in vaccinated individuals [106]. These virus specific antibodies display an oligoclonal pattern, but are not part of the main OCBs and constitute only a small fraction of intrathecally synthesized IgG [89,101]. Moreover, clonally expanded CSF B cells cultured *in vitro* were shown to display specificity for the same viruses as that described for intrathecally produced antibodies [107]. The fact that some of these antibodies are directed against RNA-viruses that most probably do not persist in the CNS, suggests that they are not a result of an ongoing virus specific immune response.

Whether the intrathecal humoral immune response in MS is pathogenic or represents an epiphenomenon has been unclear. However, there are several indications that B cells in MS may play a role in the disease process beyond their capacity to produce antibodies. Ectopic lymphoid follicles enriched with B cells and plasma cells have been observed in the meninges of patients with secondary progressive MS [108], compatible with an ongoing B cell differentiation at least in late stages of the disease. The formation of local germinal center-like structures has also been described in other autoimmune conditions [109] and may be a common feature of chronic inflammatory responses. In the case of MS, this may suggest that maturation of B cells takes place in the intrathecal compartment. This view is supported by the presence of centroblasts, a B cell population typical of secondary lymphoid organs, in the CSF [110]. In addition, antigen dependent short-lived plasma blasts are common in the CSF [94]. These observations are intriguing in light of the therapeutic potential of rituximab [111]. Rituximab targets CD20, which is carried by B cells at all stages of B cell differentiation, except for pro-B cells and plasma cells. A near complete deletion of CD20 expressing B cells in the CSF and blood was observed in treated patients, who concomitantly experienced a marked reduction in clinical attacks and a decrease in the number of lesions. Moreover, following 24 weeks of rituximab treatment the number of CD3^+^ T cells in the CSF was significantly reduced in the majority of patients [112]. However, the IgG concentration in CSF, the IgG index, the IgG synthesis rate and the number of OCBs were not affected [112]. This suggests that the therapeutic effect of rituximab is independent of antibody production and that other B cell effector functions are involved, such as bystander activation through cytokine secretion or the ability to present antigen to T cells [78].

## 4. Glatiramer Acetate in the Treatment of MS

Although the immune response in MS is predominantly detrimental to the CNS, there seems to be protective elements as well. Most treatments for MS aim at preventing lymphocyte migration, activation or proliferation, or neurotransmission [113].Glatiramer acetate (GA), one of the first-line drugs in the treatment of RRMS, provides an exceptional opportunity to study a T cell response with a proven beneficial clinical effect. Although the long term clinical efficacy of GA is questionable, GA will be discussed here as an example of immunomodulation and protective immunity in the treatment of MS. 

GA is a synthetic copolymer comprised of the four amino acids most frequent in MBP; glutamine, alanine, lysine and tyrosine. The original idea was that GA would be sufficiently identical to MBP in terms of sequence and antigenicity that it could be used as a substitute for the induction of EAE. Administration of GA unexpectedly showed the opposite effect; mice were protected against the development of clinical disease [114]. A phase III, multicenter, double blind, placebo-controlled trial, which included 251 RRMS patients, demonstrated a 29% reduction in relapse rate compared to placebo in patients who received GA for two years [115,116]. This result was the main basis for the subsequent approval by the US federal drug administration of GA for the treatment of MS. 

### 4.1. Effects on the immune system

GA binds with high affinity to HLA class II molecules of the DR isotype, and can do so without prior processing [117]. GA seems to have a number of effects on APC function, such as a reduction in the secretion of pro-inflammatory cytokines and an increase in the secretion of anti-inflammatory cytokines. This may in turn induce an anti-inflammatory phenotype in GA reactive T cells (reviewed in [118]). A GA-induced shift in the cytokine profile of GA reactive CD4^+^ T cells towards an anti-inflammatory phenotype has been demonstrated in patients by comparing the cytokine profile of GA reactive blood T cell lines from blood and CSF before and after treatment [119,120,121]. Additionally, GA may lead to anergy-induction of pathogenic lymphocytes [122,123], and restoration of function and frequency of regulatory T cells [124,125,126]. It has become increasingly clear that GA may also affect other arms of the immune system such as natural killer cells, CD8^+^ T cells and B cells [127,128,129]. 

### 4.2. Cross reactivity

Due to similarities in amino acid composition between MBP and GA, cross recognition of MBP has been thought to account for the reactivation of GA reactive T cells in the CNS. This has been based upon the observations that GA reactive T cells accumulate in the CNS of GA-treated mice [130,131] in combination with studies demonstrating cross reactivity between MBP and GA as assessed by cytokine secretion [131]. In mice, GA reactive T cells were shown to react to MBP by secretion of IL-4, IL-6 and IL-10 [132] and to target the immunodominant epitope 82–100 of MBP by T cell receptor antagonism [133]. The secretion of anti-inflammatory cytokines is thought to mediate bystander suppression of nearby pathogenic T cells within the CNS [131]. However, GA is not only efficient in MBP-induced EAE, but also in PLP and MOG-induced disease [134,135], suggesting either that cross reactivity with MBP is not essential for the therapeutic effect or that cross reactivity is a more general phenomenon. 

Studies using human T cell lines have either failed to detect cross reactivity between GA and myelin proteins [136,137] or reported it as a low frequency or unspecific event [120,121,138,139]. GA reactive T cell lines from six of seven patients displayed cross reactivity to random combinatorial peptide libraries, including peptides from MBP. Cross reactivity was determined by cytokine secretion and was consistent with a degenerative response as no dominantly cross reactive peptide emerged [120]. Two other studies reported cross reactivity to MBP by cytokine secretion in 10–25% of GA reactive cell lines [139,121]. Cross recognition assessed by proliferation was observed for three of 18 GA reactive cell lines against MBP in a patient treated with GA for 6 years [138]. Cross reactivity can, however, not be faithfully studied in polyclonal T cell lines, which may contain one T cell clone that responds to GA and another that responds to a myelin protein. In our lab, none of 20 GA-reactive CD4+ T cell clones from blood and CSF cross recognized a panel of overlapping peptides spanning the complete sequences of MBP, PLP, MOG, myelin-associated oligodendrocytic basic protein, myelin-associated glycoprotein, oligodendrocyte myelin glycoprotein, αβ-crystallin, S100β, and 2',3'-cyclic nucleotide 3'-phosphodiesterase [119]. This does not exclude that cross reactivity may occur, but rather supports that other mechanisms contribute to the efficacy of GA reactive T cell inside the CNS. 

**Figure 1:**
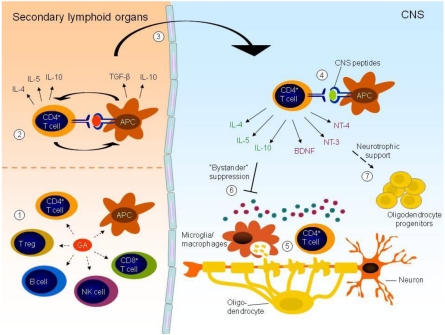
GA may affect several arms of the immune system, including CD4+ and CD8+ T cells, B cells, natural killer (NK) cells, regulatory T cells (T regs) and APCs (1). GA induces an anti-inflammatory profile in both APCs and T cells and these cell populations may reciprocally stimulate each other. The effect on CD4+ T cells is thought to involve recognition of GA presented on HLA class II molecules by APCs in peripheral lymphoid organs (2). Activated T cells may cross an inflamed BBB and gain access to the CNS (3). Within the CNS, reactivation of GA reactive T cells may occur through random cross reactivity with CNS peptides presented by CNS resident APCs (4) or in a T cell receptor independent fashion triggered by the persistent inflammatory environment within the inflamed CNS (5). Reactivation of GA reactive T cells within the CNS may lead to secretion of anti-inflammatory cytokines, which will dampen ongoing inflammation through “bystander suppression” (6). In addition, secretion of neurotrophic factors by activated T cells may affect neurogenesis and remyelination in the brain (7).

The high frequency of GA reactive T cells in the blood and CSF of treatment-naïve individuals observed by us and others, and the demonstration that the majority of these are recruited from the memory pool of T cells [121,138] suggest that GA mimics recall antigens to which the patient has been exposed previously. This is not unexpected considering the multitude of epitopes which may arise from the random composition of GA. Furthermore, the well-defined degeneracy of T cell receptors implies that a low level of random cross reactivity may be expected. Such random cross reactivity may account for reactivation of GA reactive cells in a variety of tissues and perhaps contribute to the therapeutic potential of GA in inflammatory diseases outside of the CNS [140,141]. Also, it may be hypothesized that a persistent inflammatory environment triggers a transient reactivation of GA reactive T cells in a T cell receptor independent manner, which results in release of anti-inflammatory cytokines. This may contribute to bystander suppression of local inflammation in MS and other inflammatory diseases (Figure 1).

### 4.3. Neurotrophic effect

The therapeutic effect of GA is postulated to involve neuroprotection [118,142]. GA reactive T cell lines from MS patients and healthy controls have been shown to display low basal secretion of brain-derived neurotrophic factor (BDNF), which increased upon stimulation with GA [143,144]. In mice, *in situ* secretion of BDNF by GA reactive T cells correlated with reduced neuronal damage as well as increased neuronal proliferation [145], and GA treatment was recently shown to induce remyelination in EAE mice and in a toxic model of demyelination [146,147]. Interestingly, GA has also been demonstrated to suppress the development of some models of amyotrophic lateral sclerosis, which is a neurodegenerative disease caused by mutation in the superoxide dismutase (SOD) gene with only moderate and secondary T cell infiltration [148]. It is, however, not evident that this effect is mediated by the secretion of neurotrophic factors, as SOD1 mutants also display profound immunodeficiency that could be affected by GA treatment [149]. Importantly, secretion of BDNF is not restricted to GA reactive T cells, but seems to be a more general feature of activated immune competent cells. Thus, T cells, B cells and monocytes have all been demonstrated to secrete BDNF *in vitro* and in inflammatory brain lesions [19]. This may suggest that the daily injection of GA promotes BDNF secretion as a result of a continuous activation of GA reactive peripheral T cells, which subsequently gain access to the CNS [150] (Figure 1). 

Taken together, studies in mice suggest that GA has the ability to support growth of nervous tissue [145,147,118]. To which extent this applies also in humans and the relevance in MS is less clear and needs further attention using human cells and tissue.
